# UTRN as a potential biomarker in breast cancer: a comprehensive bioinformatics and in vitro study

**DOI:** 10.1038/s41598-024-58124-5

**Published:** 2024-04-02

**Authors:** Han Li, Wenjie Zhang, Yang Liu, Zehao Cai, Ailin Lan, Dan Shu, Meiying Shen, Kang Li, Dongyao Pu, Wenhao Tan, Shengchun Liu, Yang Peng

**Affiliations:** Department of Breast and Thyroid Surgery, the First Affiliated Hospital of Chongqing Medical University, No. 1 Youyi Road, Yuzhong District, Chongqing, China

**Keywords:** Breast cancer, CIBERSORT, ESTIMATE, Prognosis, Tumor microenvironment, UTRN, Cancer, Cell biology, Drug discovery, Biomarkers, Oncology

## Abstract

Utrophin (UTRN), known as a tumor suppressor, potentially regulates tumor development and the immune microenvironment. However, its impact on breast cancer’s development and treatment remains unstudied. We conducted a thorough examination of UTRN using both bioinformatic and in vitro experiments in this study. We discovered UTRN expression decreased in breast cancer compared to standard samples. High UTRN expression correlated with better prognosis. Drug sensitivity tests and RT-qPCR assays revealed UTRN’s pivotal role in tamoxifen resistance. Furthermore, the Kruskal–Wallis rank test indicated UTRN’s potential as a valuable diagnostic biomarker for breast cancer and its utility in detecting T stage of breast cancer. Additionally, our results demonstrated UTRN’s close association with immune cells, inhibitors, stimulators, receptors, and chemokines in breast cancer (BRCA). This research provides a novel perspective on UTRN’s role in breast cancer’s prognostic and therapeutic value. Low UTRN expression may contribute to tamoxifen resistance and a poor prognosis. Specifically, UTRN can improve clinical decision-making and raise the diagnosis accuracy of breast cancer.

## Introduction

Female breast cancer is the most commonly diagnosed cancer, presenting a substantial threat to women’s health^1^. Approximately 70% of breast cancer patients exhibit estrogen receptor-positive subtypes, where the estrogen receptor significantly contributes to breast cancer development^2^. First-line treatment for estrogen receptor-positive patients involves endocrine therapy alongside surgery. Tamoxifen is a commonly used endocrine drug in the treatment of estrogen receptor-positive breast cancer^3,4^, but metastasis or recurrence still occurs in 30–40% of patients after treatment, resulting in tamoxifen resistance^5,6^. Many studies have investigated potential mechanisms of tamoxifen resistance, categorizing it into primary and acquired forms^6^, possibly linked to transcription factors, autophagy, and cell cycle regulators^7^.

The tumor immune microenvironment (TME) comprises a diverse array of cell types within solid tumors, including tumor cells, immune cells, tissue-specific residents, and recruited stromal cells^8,9^. The growth of malignancies is aided by this tumor heterogeneity, and endocrine resistance may be significantly influenced by tumor-associated macrophages or T lymphocytes that have infiltrated the TME^10^. Immune checkpoint blockade therapy (ICBT) has advanced cancer treatment, supported by recent evidence. Additionally, within the tumor microenvironment, estrogen receptor-associated signaling may potentially exert immunomodulatory effects^11^. However, the relationship between the tumor microenvironment and the response to tamoxifen treatment has not been extensively reported.

Utrophin (UTRN), encoding a component of the cytoskeleton, resides on chromosomal band 6q24^12,13^. UTRN functions as a tumor suppressor gene and impacts the growth of various malignancies. Recent research has shown that high UTRN expression suppresses the proliferation of melanoma^14^, while its expression is reduced in melanoma, lung, and colon cancers^13^. Moreover, UTRN downregulation has been observed in breast cancer, with in vitro UTRN overexpression inhibiting tumor cell development^13,15^. However, UTRN’s function in the response to endocrine therapy and its role in the tumor immune microenvironment in breast cancer remain unexplored.

In this study, we identified the differential gene UTRN in the drug resistance datasets and confirmed its important role in tamoxifen sensitivity. We also evaluated its immunological associations and explored downstream pathways. Using the CIBERSORT method, we calculated the proportion of immune and stromal cells in BRCA samples from The Cancer Genome Atlas (TCGA) database. Given the recent focus on ceRNA networks, we uncovered the regulatory ceRNA network of UTRN to investigate potential mechanisms. This study highlights UTRN as a prognostic and predictive biomarker for endocrine therapy in breast cancer, establishing a link with the immunological microenvironment.

## Materials and methods

### TCGA and GEO data collection

The Cancer Genome Atlas Program (TCGA) provided the clinical information, HTSeq-FPKM transciptome information, and miRNA information for breast invasive carcinoma (The Cancer Genome Atlas Program—National Cancer Institute). The mRNA expression for tamoxifen-resistant databases, such as GSE9893 (n = 155), GSE159968 (n = 9), and GSE125738 (n = 6), were downloaded from the Gene Expression Omnibus database (GEO, https://cancergenome.nih.gov). The description about GSE datasets were shown in Supplementary table 1. Three databases’ differentially expressed genes were combined to discover key genes in breast cancer endocrine therapy. These genes were expressed differently in tamoxifen-resistant and tamoxifen-sensitive cells.

### Identification of differentially expressed tamoxifen-resistant genes

Ten genes were retrieved from intersecting three tamoxifen treatment databases. The “limma” package of R was applied to identify differentially expressed genes (DEGs) between tamoxifen sensitive and resistant samples with *p*-value < 0.05 and fold change (FD) > 1. Volcano plots were plotted with the R package.

### Diagnostic and survival value analysis of UTRN

The TCGA-BRCA datasets were used to download the clinical data of the patients, and the Kruskal–Wallis rank sum test was used to examine the association between UTRN expression and specific cancer stages, TNM, age, and gender. To evaluate the prognostic importance of clinical characteristics, the R package “rms” was used to combine data and create nomograms using the Cox method. The *P* value < 0.05 suggested that UTRN was related to this clinical feature.

### Gene set enrichment analysis (GSEA)

GSEA was utilized to investigate the roles of UTRN. Firstly, R language was used to get the expression data of mRNA gene sets. According to the median expression of the target gene, we separated BRCA patients into low expression and high expression groups. Then, GSEA _4.1.0 analytic program was employed. The target sets for GSEA, which was conducted using the software gsea-3.0 acquired from Broad Institute, were the Hallmark and C7 gene sets v6.2 collections, which were obtained from the Molecular Signatures Database. Gene sets deemed significant were those with NOM *p* < 0.05 and FDR q < 0.06.

### Cell culture and transfection

Human ER-positive BC cells (MCF7 and T47D) were cultured in medium supplemented with high-glucose DMEM (Gibco, Thermo Fisher Scientific, Waltham, MA, USA) containing 10% foetal bovine serum (FBS) (ExCell Bio) in a humidified incubator with 5% CO2 at 37 °C. All siRNA fragments were purchased from Tsingke Biological Technology. The primer target sequences of siUTRN-1 and siUTRN-2 were CTCTTAGAGTTGAGTACAA and CTGTGGATGATCGCCTTAA, respectively. SiRNA transfections were conducted using Lipofectamine 2000 (Thermo Fisher Scientific, Waltham, MA, USA), following standard protocols in accordance with the manufacturer’s guidelines (number of replicates = 3).

### RNA isolation, reverse-transcription reaction and quantitative real-time PCR (qPCR)

Total RNA was extracted from cultured cells using the Total RNA Extraction Kit (Tiangen, Beijing, China), and reverse transcription was conducted using a 4xRT mix (MedChemExpress, Shanghai, China). The PCR primers used were as follows: UTRN F, 5′- CTGTGGATGATCGCCTTAAA-3′, and R, 5′-CTGGACTGACGTAGAGAGAA-3′. Quantitative RT–PCR was carried out in a 10-μL PCR reaction using SYBR Premix Ex TaqTM II (MedChemExpress) on a Bio-Rad CFX96 Real-Time PCR System (Bio-Rad Laboratories, Inc., Hercules, CA, USA). The procedure involved an initial cycle of 2 min at 95 °C, followed by 39 cycles at 95 °C for 30 s, a cycle of 30 s at 58 °C, and a cycle of 20 s at 72 °C. Three independent experiments were conducted per group. Relative gene expression was normalized to GAPDH and assessed using the 2^−ΔΔCt^ method (number of replicates = 3).

### Drug sensitivity assay

Cells were seeded in 96-well plates at a density of approximately 4000 cells/100 µL per well and incubated for 24 h. Subsequently, they were exposed to different concentrations of 4-OH tamoxifen (0, 2, 4, 8, 16, 32 μM) for 72 h. Cell viability was determined using Cell Counting Kit-8 (CCK-8) reagent (MedChemExpress, Shanghai, China). Following a 2-h incubation, the absorbance was measured at 450 nm. The ratio of the absorbance corresponding to each drug concentration to the absorbance without drug addition is the cell viability under the influence of 4-OH tamoxifen. *p* < 0.05 was considered a statistically significant difference in drug sensitivity.

### Correlation of UTRN expression with immune cell infiltration

Employing the CIBERSORT method, we determined the proportions of 22 infiltrating immune cells. We assessed the correlation between immune cell infiltration and UTRN expression levels using the Pearson correlation test. Subsequently, the Wilcoxon rank test was applied to identify immune cells that exhibited significant differences between the groups with high and low UTRN expression. Statistical significance was set at a *P* value of 0.05 or lower.

### TIMER database analysis

The Tumor Immune Estimation Resource (TIMER2.0) (https://timer.cistrome.org/) is a database used to examine the amounts of diverse gene expression and immune cells that infiltrate tumors in various cancer types. By using gene modules, we evaluated UTRN expression in diverse malignancies and its association with tumor-infiltrating lymphocytes (TILs). *p* < 0.05 was considered a statistically significant correlation.

### TISIDB database analysis

To examine the connection between cancers and the immune system, TISIDB integrated a variety of heterogeneous data sources (http://cis.hku.hk/TISIDB/index.php). The database can help researchers find new immunotherapy targets, forecast the effectiveness of immunotherapy, and learn more about the interactions between cancers and immune cells. In the current investigation, we used the TISIDB database to evaluate the connections between UTRN and 28 TILs, 24 immunoinhibitors, 40 immunostimulators, 41 chemokines, and 18 receptors in BRCA. *p* < 0.05 was considered a statistically significant correlation.

### CeRNA network prediction

Utilizing the website ENCORI (http://starbase.sysu.edu.cn/) (CLIP-Data >  = 1,programNum >  = 2), the target miRNAs for UTRN were predicted. Additionally, we looked at the relationship between miRNA expression and UTRN expression to choose the miRNAs that were more suitable for building the ceRNA network. Then, a subset of miRNAs relevant to survival were identified and used to create ceRNA networks. Using the ENCORI website, the target lncRNAs of miRNAs were also predicted, and lncRNAs that had a negative correlation with the expression of particular miRNAs were taken out. For the creation of ceRNA networks, specific lncRNAs that were associated with survival were employed.

### CeRNA network construction

The ceRNA network construction was created using Cytoscape 3.6.1.

### Statistical analysis

All data analysis and visualizations were performed using R (3.6.3). The UTRN expression was analyzed in unpaired samples using Wilcoxon rank-sum test, while paired samples were analyzed using Wilcoxon signed-rank test. Cox regression analysis and Kaplan–Meier analysis were performed to assess the prognostic factors. Using multivariate Cox analysis, we compared the impact of UTRN expression on survival and other clinical characteristics. We used the median UTRN expression as the cut-off point to perform GSEA analysis. GraphPad Prism 8.0 software (San Diego, CA, USA) was used for data analysis. Student’s t-test or one-way ANOVA was used to analyse differences between groups. In all statistical analyses, *P*-values below 0.05 indicated statistical significance.

## Results

### Result 1. Analysis process of this study

**Figure 1:**
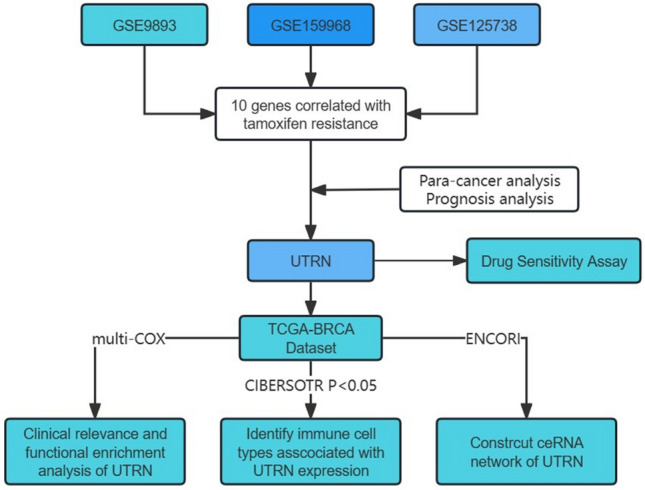
Analysis workflow of this study.

Figure 1 illustrates the analysis procedure of our study. Transcriptome RNA-seq data from three databases were obtained from the GEO database to identify functional genes in tamoxifen-resistant BRCA samples. Following the evaluation of the predictive significance of 10 DEGs, our focus shifted to UTRN for subsequent analyses. These analyses encompassed survival and clinicopathological characteristic correlation assessments, COX regression, Gene Set Enrichment Analysis (GSEA), and correlation with TILs.

### Result 2. Identification of differentially expressed genes in tamoxifen resistance database

**Figure 2:**
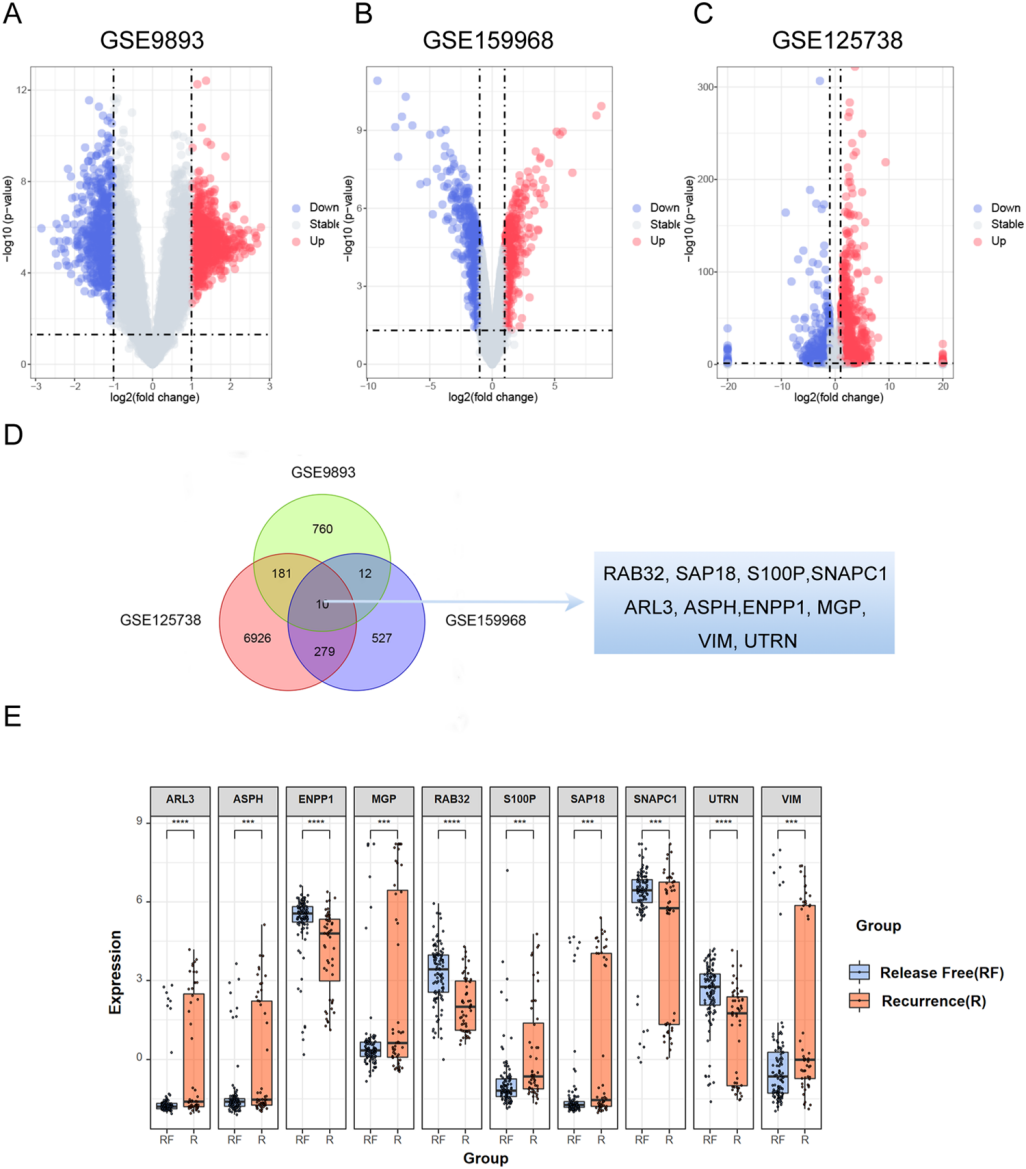
Identification of tamoxifen-resistance related genes in GEO databases. (**A**–**C**) Volcano plot of clinical recurrence and survival specimen data set GSE9893, tamoxifen-sensitive and tamoxifen-resistance ER (estrogen receptor) positive cell lines GSE159968 and GSE125738. DEGs were detected by Wilcoxon rank sum test (q < 0.05 & |log2FC|> 1). (**D**) Venn graph of differentially expressed genes in intersection of three GEO databases. (**E**) The expression of differential expressed genes in clinical sample data sets GSE9893 (Wilcoxon-Mann–Whitney test, ***p < 0.001; ****p < 0.0001).

We analyzed standardized gene expression levels in primary tumors from 155 patients using microarray dataset GSE9893 (Fig. 2A). Additionally, we obtained gene expression data for the breast cancer cell lines MCF7 (tamoxifen-sensitive) and MCF7R (tamoxifen-resistant), as well as T47D (tamoxifen-sensitive) and T47DR (tamoxifen-resistant), from the microarray datasets GSE159968 (Fig. 2B) and GSE125738 (Fig. 2C) in the GEO database, respectively. By combining the analysis of these datasets, we identified 10 genes as potential candidates for tamoxifen resistance, including RAB32, SAP18, S100P, SNAPC1, ARL3, ENPP1, MGP, VIM, and UTRN (Fig. 2D). Figure 2E illustrates the differential expression levels of DEGs in clinically resistant samples.

### Result 3. Expression and survival value analysis of UTRN

**Figure 3:**
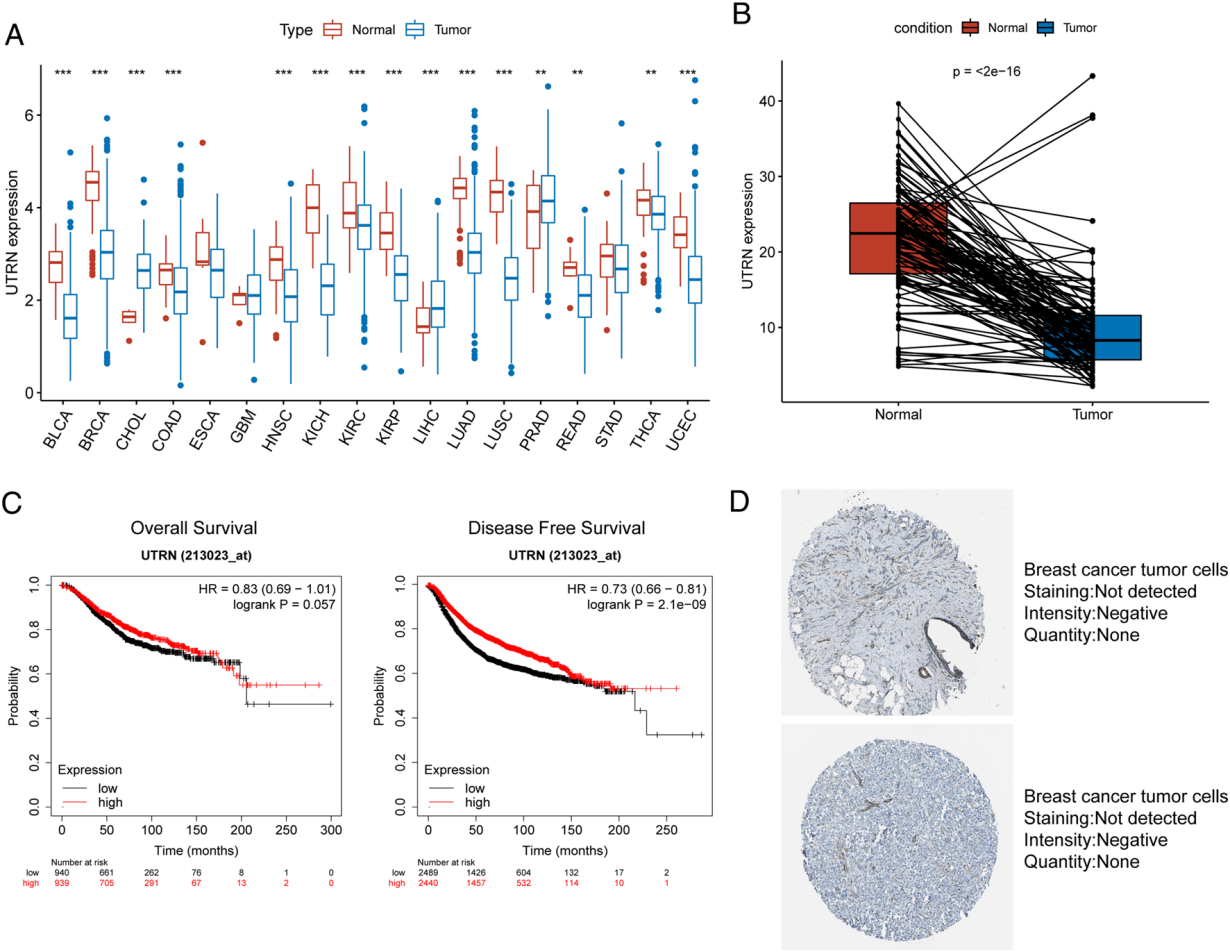
mRNA and protein expression level of UTRN in BRCA from TCGA database. (**A**) The UTRN expression in different cancer types from the TCGA databases. (**p < 0.01; ***p < 0.001); (**B**) The expression level of UTRN was significantly down-regulated in BRCA tissues (paired T test, *p* < 0.001). (**C**) Survival analysis of UTRN in TCGA-BRCA database from Kaplan–Meier Plotter. (**D**) Representative immunohistochemistry images of UTRN in BRCA derived from the Human Protein Altas website.

Through a paired analysis of invasive breast cancer (BRCA) tissues, we observed that UTRN expression was significantly lower compared to that in normal breast tissues. Pan-cancer analysis of the TCGA database indicated that the expression level of UTRN was lower in most cancer tissues compared to normal tissues, as determined by t-test (Fig. 3A,B). Investigation on the Kaplan–Meier Plotter website revealed that patients with high UTRN expression experienced better overall survival and progression-free survival than those with low UTRN expression (Fig. 3C). To further investigate the differences in UTRN expression between cancer and normal tissues, we searched for immunohistochemical data from tumor samples on The Human Protein Atlas website. We found that UTRN was not detected in breast cancer tumor tissues (Fig. 3D). UTRN expression was reduced in tumor samples, and its overexpression predicted a better prognosis.

### Result 4. Knockdown of UTRN induces tamoxifen resistance in HR + BC cells

**Figure 4:**
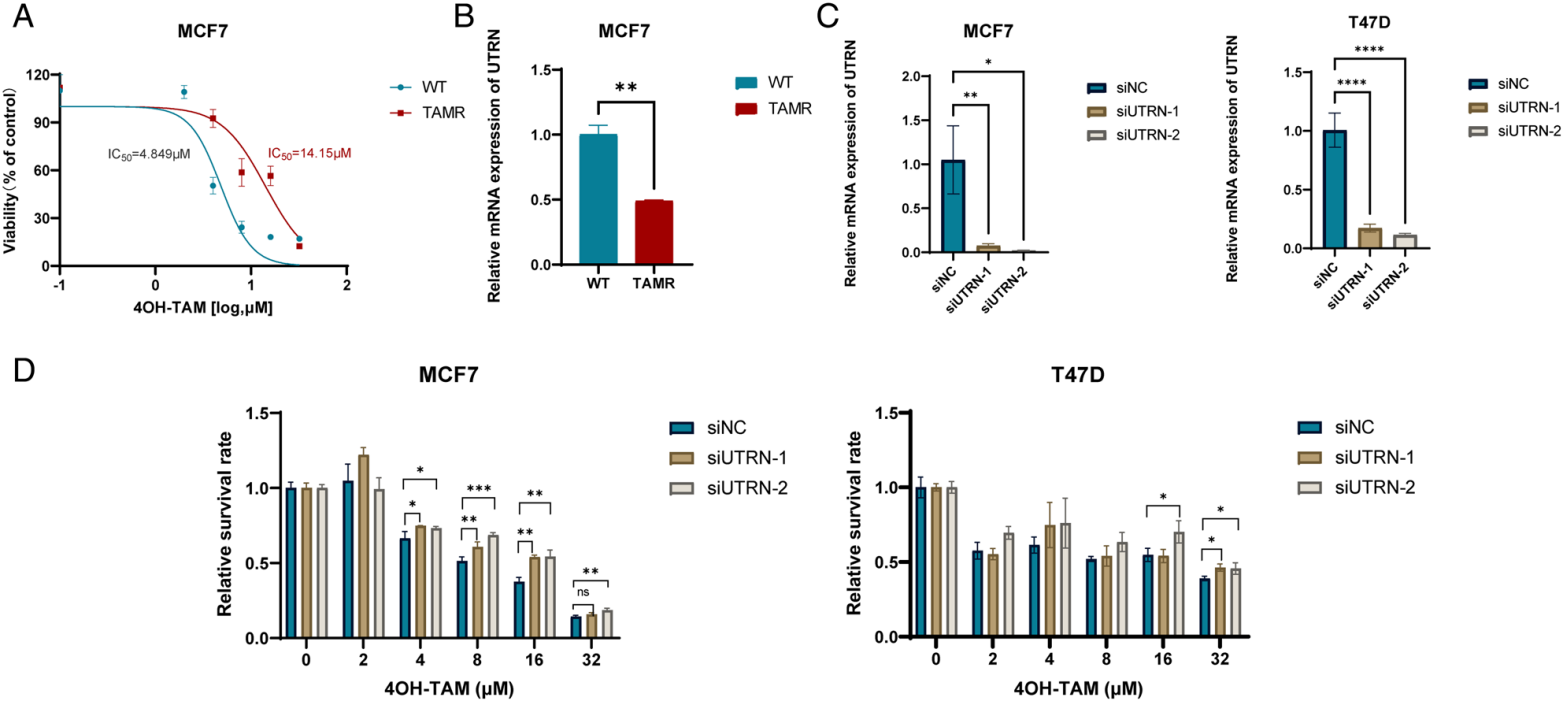
UTRN low expression reduces tamoxifen sensitivity of HR + breast cancer cells. (**A**) 4OH-tamoxifen sensitivity of MCF-7 wild type (WT) and tamoxifen resistant MCF-7 (TAMR); (**B**) UTRN mRNA expression in MCF-7 wild type (WT) and tamoxifen resistant MCF-7 (TAMR); (**C**) UTRN mRNA expression in negative control (siNC), siUTRN-1 and siUTRN-2 of MCF-7 and T-47D cells; (**D**) Effect of different doses of 4OH-TAM on negative control (siNC), siUTRN-1 and siUTRN-2 of MCF-7 and T-47D cells after 48h of treatment respectively. Relative RNA levels were quantified via qRT-PCR, GAPDH was used for normalization. All data are expressed as mean ± SEM. Statistical significance was determined by two-tailed Unpaired Student’s t test (**B** and **C**) or one-way ANOVA followed by Dunnett’s multiple comparisons test (**D**). *P* values are indicated (ns: not significant, *p < 0.05; **p < 0.01; ***p < 0.001; ****p < 0.0001).

To validate biological function of UTRN in HR + BC cells, we constructed tamoxifen-resistant (TAMR) MCF-7 cells. The CCK-8 assay confirmed that tamoxifen-resistant MCF-7 cells exhibited an IC50 of 14.15 M, which was three times higher than that of tamoxifen-sensitive MCF-7 cells (Fig. 4A). We then performed RT-qPCR to measure the mRNA levels of UTRN in tamoxifen-sensitive cells and resistant cells. Compared with MCF-7 wild type (WT), the expression of UTRN was lower in tamoxifen-resistant MCF-7 cells (TAMR) (Fig. 4B). RT-qPCR was used to confirm the knockdown efficiency (Fig. 4C). Concurrently, CCK-8 tests demonstrated that UTRN knockdown reduced the sensitivity to tamoxifen in HR + breast cancer cells (Fig. 4D).

### Result 5. Prognostic value of UTRN

**Figure 5:**
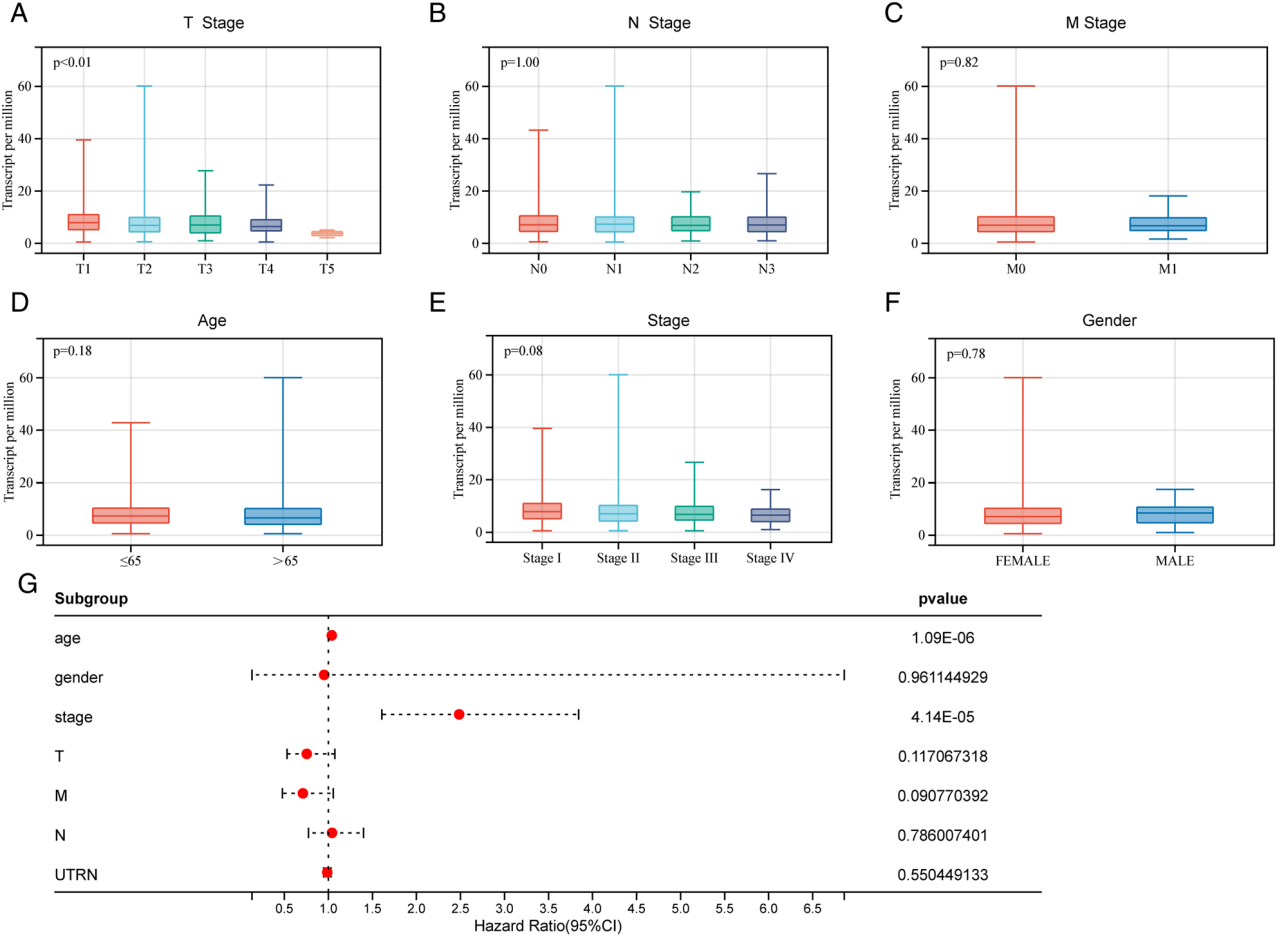
UTRN expression of BRCA according to different clinical characteristics. (**A**) T stage (Kruskal–Wallis); (**B**) N stage (Kruskal–Wallis); (**C**) M stage (Wilcoxon-Mann–Whitney test); (**D**) age (Wilcoxon-Mann–Whitney test); (**E**) stage (Kruskal–Wallis); (**F**) gender (Wilcoxon-Mann–Whitney test). (**G**) Nomograms were established using the Cox method, and the prognostic significance of these features in 1023 samples was assessed.

We obtained clinical data from the TCGA-BRCA database to gain a better understanding of the role of UTRN expression in BRCA progression. Subsequently, using the Wilcoxon-Mann–Whitney test or the Kruskal–Wallis rank test, we explored potential correlations between UTRN expression and the clinical characteristics of tumors (Fig. 5A–F). Clinical information corresponding to Fig. 5 is included in Supplementary table 2. The results indicated that higher T stages (Fig. 5A, *p* < 0.01) and pathological stages (Fig. 5E, *p* = 0.08) were significantly connected with reduced UTRN expression. We utilized the R package rms to integrate data on survival time, survival status, age, gender, stage, TNM, and UTRN expression. Nomograms were created using the Cox method to evaluate the predictive importance of these parameters in 1023 individuals (excluding samples with missing data) (Fig. 5G). Overall, these findings showed that the pathologic stage and advanced T stage of the BRCA gene were clearly linked with reduced expression of UTRN.

### Result 6. Gene set enrichment analysis of UTRN

**Figure 6:**
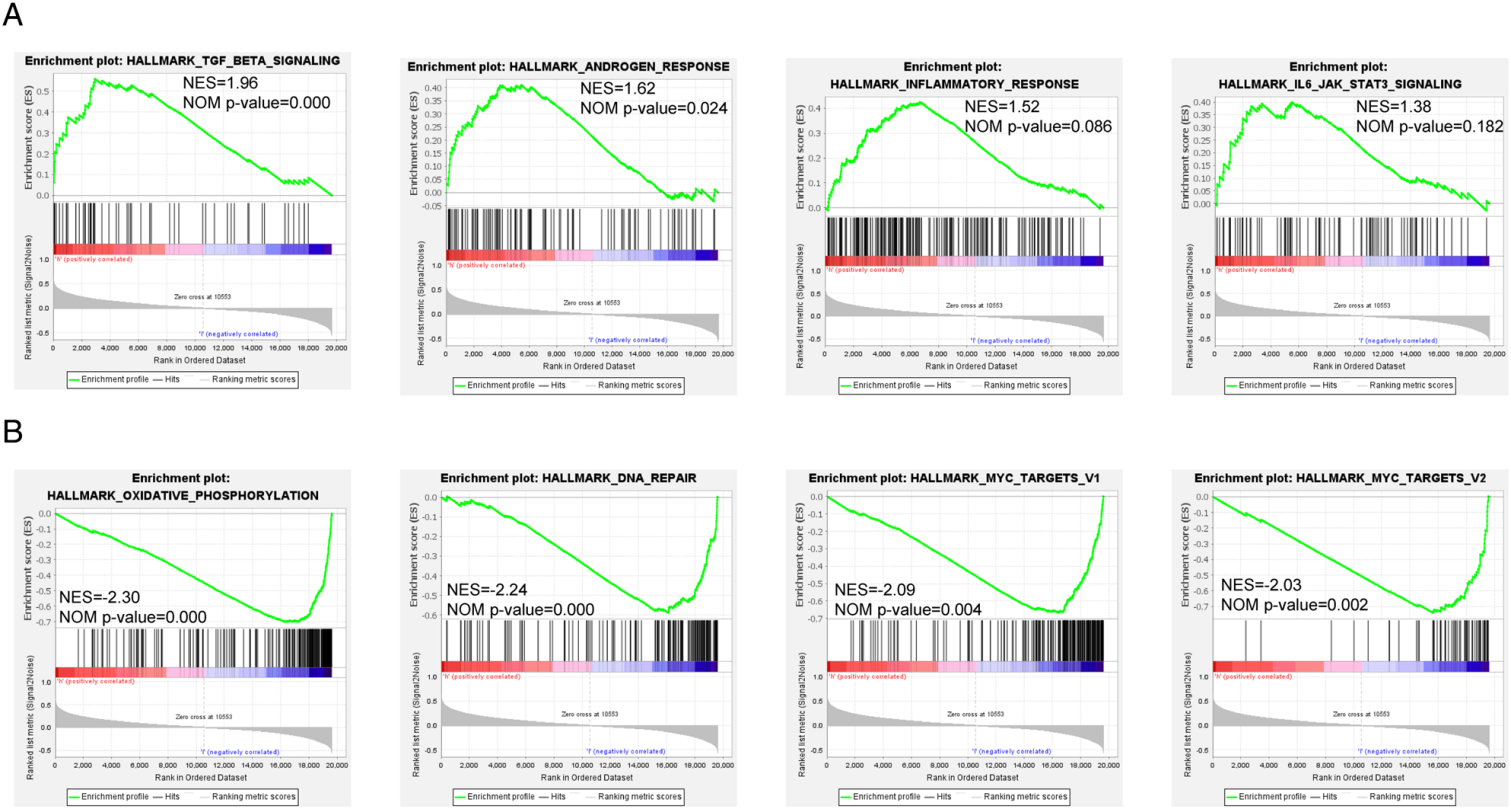
Gene set expression analysis of UTRN expression. (**A**) The major enriched pathways for high-expression group. (**B**) The major enriched pathways for low-expression group. NOM *p* < 0.05, FDR q < 0.25, and |NES|> 1 are set as the significance threshold.

With 1000 permutations, we examined the potential function of UTRN using gene set enrichment software (version 4.1.0). Patients were divided into ‘high’ and ‘low’ groups based on the appropriate cutoff value for UTRN expression data from the TCGA database. To identify the critical pathways associated with tamoxifen resistance in the groups with low vs. high UTRN gene expression levels, we conducted Kyoto Encyclopedia of Genes and Genomes (KEGG) analysis (c2.cp.kegg.v7.4.symbols.gmt). The UTRN highly expressed group showed enrichment in TGF- signaling, androgen response, inflammatory response, and the IL6-JAK STAT3 signaling pathway. Conversely, we observed downregulation of oxidative phosphorylation, DNA repair, and MYC pathways in the UTRN low expression group (Fig. 6A,B).

### Result 7. UTRN correlates with immune infiltration in BRCA microenvironment

**Figure 7:**
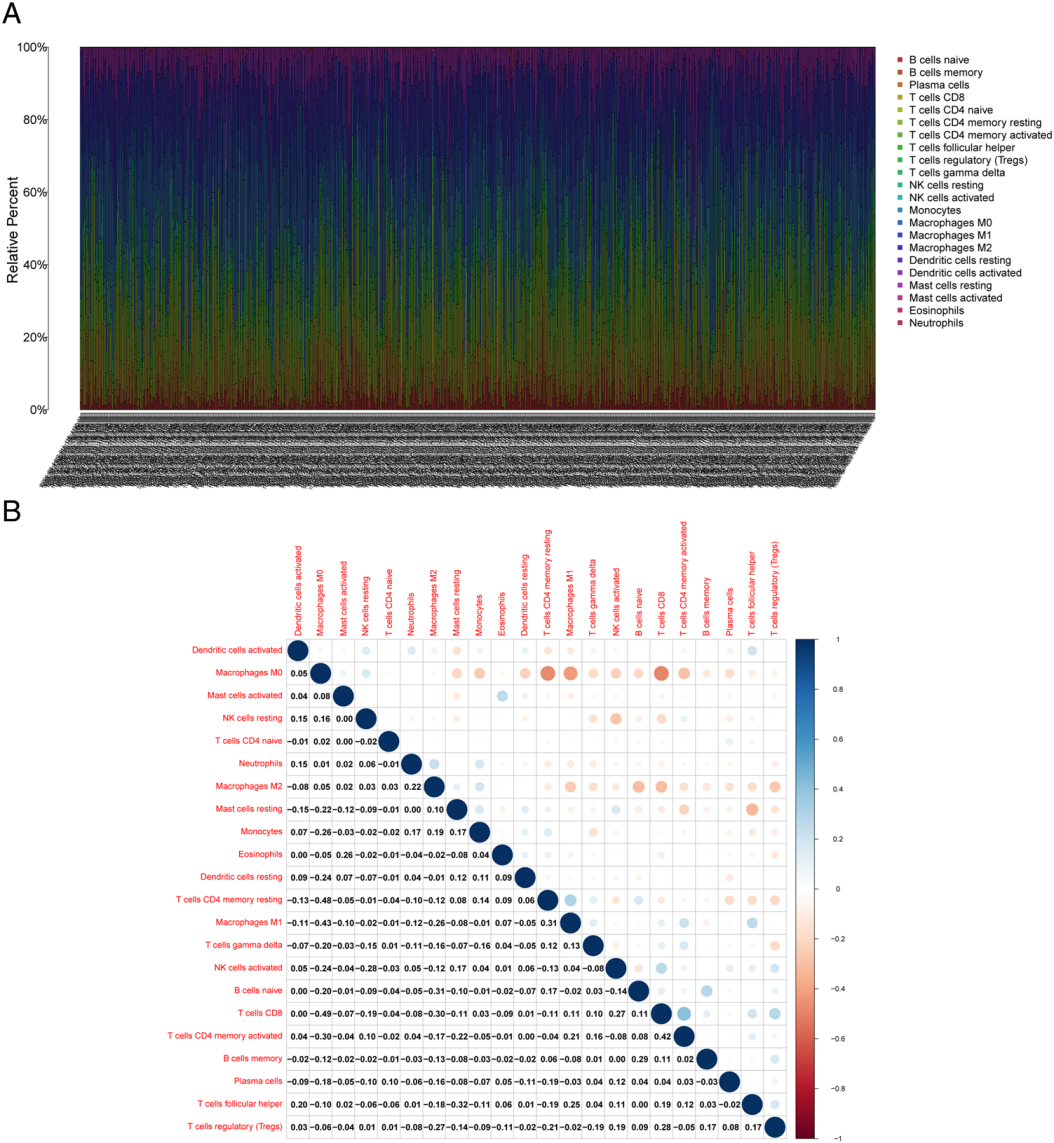
TIC profile in BRCA tumor samples and correlation analysis. (**A**) Barplot displaying the distribution of the 21 different TIC types in BRCA tumor tissues. Sample IDs appeared in the plot’s column names. (**B**) Heatmap displaying the correlation between 21 different TIC types with numerical values in each tiny box indicating the *p* value of the correlation between two different cell types. The Pearson coefficient was used for the significance test, and the color of each tiny color box represented the corresponding correlation value between two cells.

**Figure 8:**
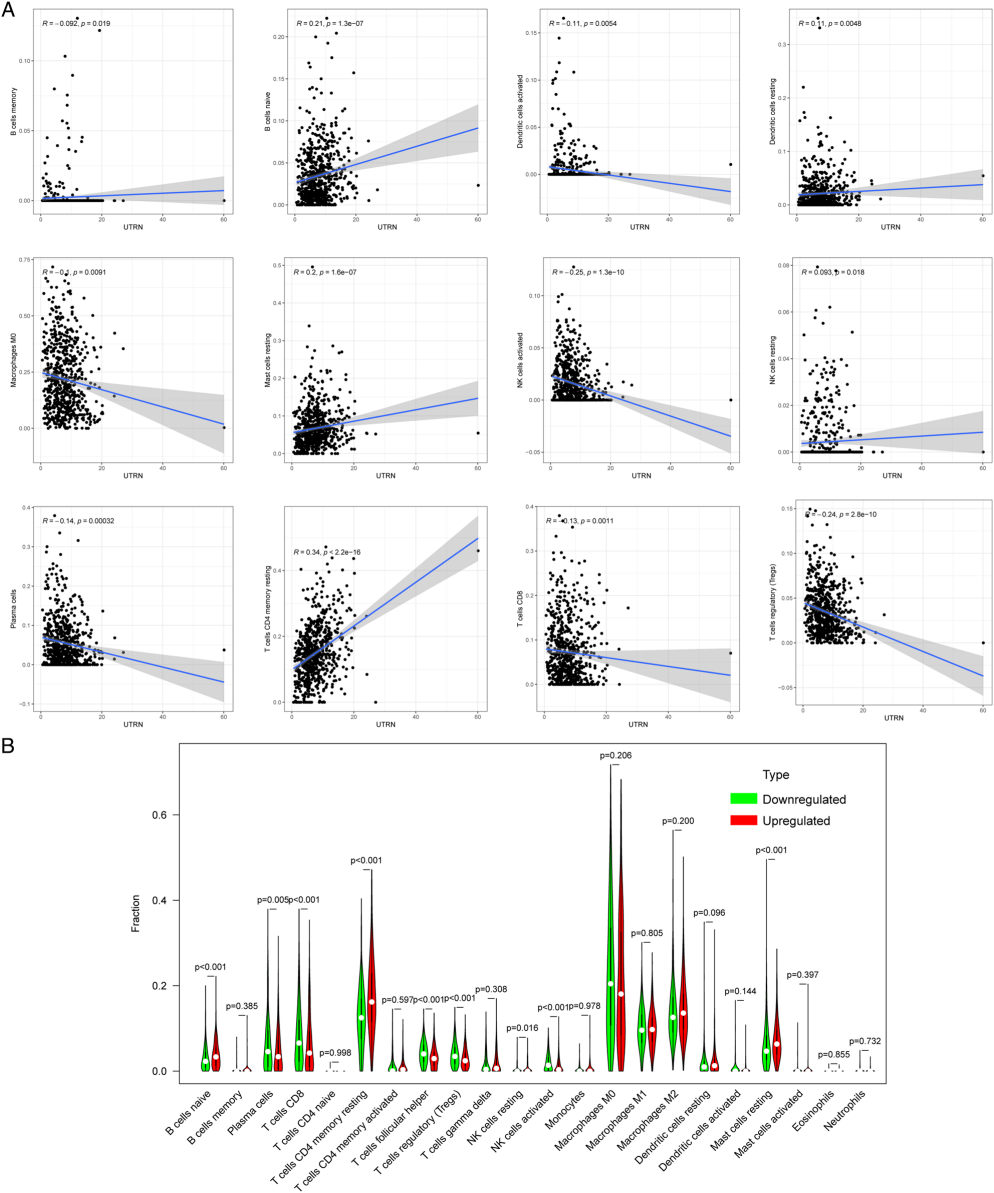
Correlation of TICs proportion with UTRN expression. (**A**) Wilcoxon rank sum was performed to determine whether the ratio differentiation of 21 different immune cell types between BRCA tumor samples with low or high UTRN expression relative to the median of UTRN expression level was statistically significant. (Spearman, *p* < 0.05 regarded statistically significant) (**B**) Data from the TIMER2.0 database show a correlation between UTRN expression and infiltration levels of memory B cells, naive B cells, macrophage M0, mast cells at rest, NK cells activated, NK cells at rest, plasma cells, CD4 + T cells memory resting, CD8 + T cells, and T-cell regulatory in breast cancer. Online, color photos are accessible.

Based on previous gene enrichment analyses, UTRN expression may be associated with the tumor immune microenvironment (TME). We employed the CIBERSORT algorithm to detect 22 immune cell types in the BRCA microenvironment, further exploring UTRN’s predictive role in TME (Fig. 7A). Additionally, we used Pearson analysis to unveil patterns among various immune cells (Fig. 7B). To assess the correlation between UTRN expression and immune infiltration, we relied on the online database TIMER 2.0. Using Spearman rank correlation analysis. And we observed that UTRN expression was significantly negatively correlated with activated dendritic cells, macrophages M0, NK cells, plasma cells, CD8 + T cells, and regulatory T cells (Tregs) (Fig. 8A). Furthermore, we conducted the Wilcoxon-Mann–Whitney test, revealing that the fractions of naïve B cells, memory B cells, resting memory CD4 + T cells, and activated NK cells in the UTRN low-expression group were relatively lower than those in the UTRN high-expression group. Conversely, plasma cells, CD8 + T cells, follicular helper T cells, regulatory T cells (Tregs), M0 macrophages, and resting mast cells were statistically higher in the UTRN low-expression group (Fig. 8B).

### Result 8. Immunoregulators and UTRN expression are linked in breast cancer

Immunomodulators and UTRN expression are linked in breast cancer. Immunomodulators are crucial elements that have an impact on how well the immune system works. This study showed a strong relationship between UTRN and immunoinhibitory factors (*p* < 0.05), such as CD96 (rho = 0.155), CD160 (rho = 0.11), CD244 (rho = 0.056), CD274 (rho = 0.282), CSF1R (rho = 0.215), HAVCR2 (rho = 0.12), IL10RB (rho = − 0.065), KDR (rho = 0.452), LAG3 (rho = − 0.235), LGALS9 (rho = − 0.093), PDCD1 (rho = − 0.101), PDCD1LG2 (rho = 0.207), PVRL2 (rho = − 0.122), TGFBR1 (rho = 0.393) and VTCN1 (rho = − 0.129) (Supplementary Fig. 1A). The UTRN level was also strongly related with immunostimulatory factors (*p* < 0.05), including C10orf54 (rho = 0.07), CD28 (rho = 0.208), CD40LG (rho = 0.153), CD70 (rho = − 0.121), CD80 (rho = 0.119), CD86 (rho = 0.103), CXCL12 (rho = 0.389), CXCR4(rho = 0.082), ENTPD1 (rho = 0.4), ICOSLG (rho = − 0.102), IL2RA (rho = 0.059), IL6 (rho = 0.083), IL6R (rho = 0.346), MICB (rho = 0.078), NT5E (rho = 0.357), NT5E (rho = 0.357), PVR (rho = − 0.247), RAET1E (rho = 0.159), TMEM173 (rho = 0.129), TNFRSF4 (rho = − 0.295), TNFRSF9 (rho = 0.166), TNFRSF14 (rho = − 0.247), TNFRSF17 (rho = 0.067), TNFRSF18 (rho = − 0.304), TNFRSF25 (rho = 0.32), TNFSF4 (rho = 0222), TNFSF9 (rho = − 0.107), TNFRSF13B (rho = 0.184), TMIGD2 (rho = 0.507), TNFSF13B (rho = 0.184), TNFSF14 (rho = 0.187) and TNFSF15 (rho = 0.375) (Supplementary Fig. 1B). Therefore, UTRN is directly involved in modulating immune interactions, which may help tumor immune escape when UTRN expression is low.

### Result 9. Chemokines and UTRN expression are linked in breast cancer

Chemokines are essential for controlling immune cell invasion. We investigated the connections between UTRN, 41 chemokines, and 18 BRCA receptors. This study found a connection between UTRN expression and chemokine expression (*p* < 0.05). UTRN expression was significantly linked with CCL3 (rho = − 0.084), CCL5 (rho = − 0.077), CCL8 (rho = − 0.068.), CCL14 (rho = 0.226), CXCL17 (rho = − 0.06), CCL18 (rho = − 0.081), CCL20 (rho = 0.49.), CX3CL1 (rho = 0.448), CXCL2 (rho = 0.513), CXCL13 (rho = 0.468), CXCL11 (rho = − 0.131), CCL21 (rho = 0.12), CCL22 (rho = 0.115), CCL28 (rho = 0.161), CXCL3 (rho = − 0.074), CXCL9 (rho = 0.07), CXCL12 (rho = 0.389), CXCL13 (rho = − 0.085), CXCL14 (rho = 0.144), CXCL16 (rho = − 0.066) and CXCL17 (rho = 0.077) (Supplementary Fig. 2A). In addition, we found significant correlations between UTRN expression and chemokine receptors (p < 0.05), including CCR1 (rho = 0.128), CCR2 (rho = 0.224), CCR4 (rho = 0.352), CCR5 (rho = 0.146), CCR6 (rho = 0.17), CCR8 (rho = 0.166), CCR10 (rho = − 0.171), CX3CR1 (rho = 0.403), CXCR1 (rho = 0.122), CXCR2 (rho = 0.302), CXCR4 (rho = 0.082) and CXCR6 (rho = 0.072) (Supplementary Fig. 2B). These findings also suggest that UTRN may function as an immunoregulatory factor in breast cancer.

### Result 10. UTRN-related ceRNA network construction in BRCA

**Figure 9:**
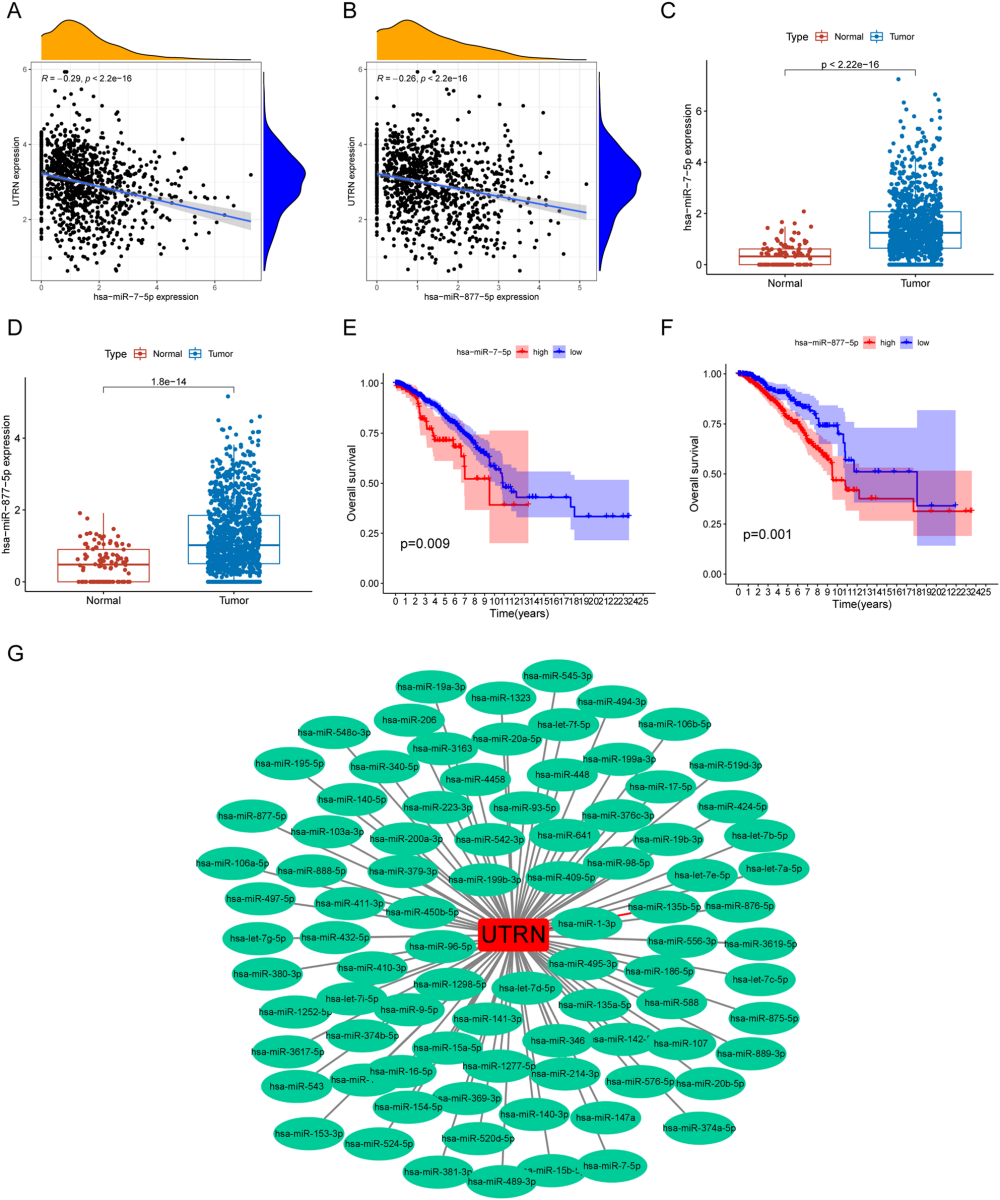
UTRN-related miRNAs in BRCA. (**A**, **B**) hsa-miR-7-5p and hsa-miR-877-5p, possible target miRNAs for UTRN through the prediction of ENCORI database, is down-expressed in BRCA from TCGA cohort. Hsa-miR-7-5p and hsa-miR-877-5p expression was negatively correlated with UTRN expression. Correlation test is conducted by the Pearson coefficient. *p*-value < 0.01 is the significance threshold. (**C**, **D**) The expression of hsa-miR-7-5p and hsa-miR-877-5p in human normal compared with that in breast cancer patients from TCGA-BRCA database (Wilcoxon-Mann–Whitney test). (**E**, **F**) Patients with low hsa-miR-7-5p and hsa-miR-877-5p expression were closely correlated with better overall survival (two-side log-rank test). (**G**) Forest map of possible miRNAs of UTRN.

**Figure 10:**
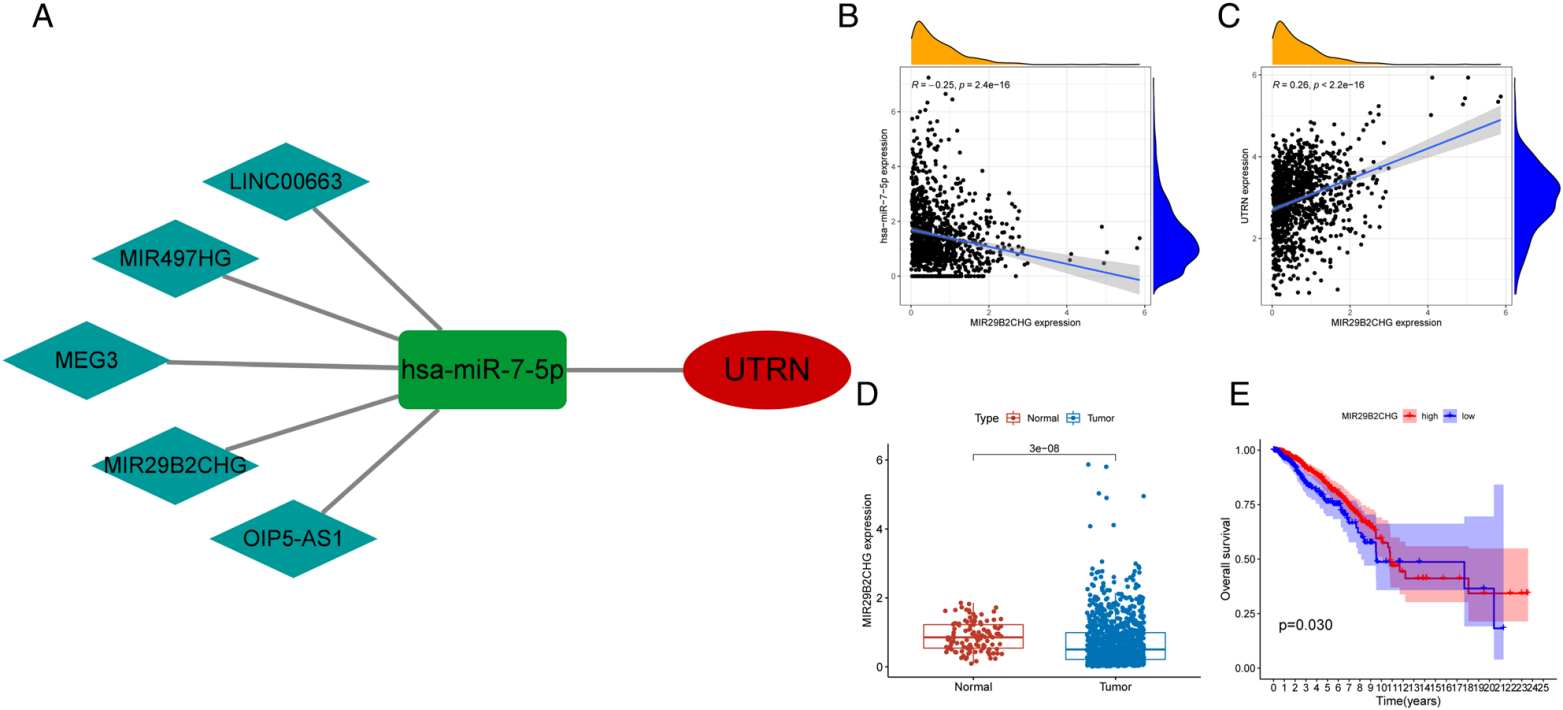
UTRN-related ceRNA network construction in BRCA. (**B**, **C**) MIR29B2CHG expression was negatively correlated with hsa-miR-7-5p expression and positively correlated with UTRN expression. Correlation test is conducted by the Pearson coefficient. *p*-value < 0.01 is the significance threshold. (**D**) MIR29B2CHG, a possible target lncRNA for hsa-miR-7-5p through the prediction of ENCORI database, is low-expressed in BRCA from TCGA cohort (Wilcoxon-Mann–Whitney test). (**E**) Patients with lower MIR29B2CHG expression were closely correlated with poorer overall survival (two-side log-rank test).

Through the various analyses above, we found that UTRN predicts prognosis value, tamoxifen resistance, and the associated functions of the tumor immune microenvironment in breast cancer. In order to explore the upstream regulation of UTRN, we focused on the establishment of ceRNA networks. We examined the predicted target genes from ENCORI database by correlation analysis (Supplementary table 3). There were 12 microRNAs negatively correlated with UTRN (Fig. 9A,B and Table 1)). The expression level of hsa-miR-7-5p and hsa-miR-877-5p was significantly higher in tumor than that in normal tissue (Fig. 9C,D). Survival analysis revealed that poor prognosis value of highly expressed hsa-miR-7-5p (*p* = 0.009) and hsa-miR-877-5p (*p* = 0.001) (Fig. 9E,F). Besides, we applied cytoscape software (version 3.9.0) to draw the forest map of UTRN and its correlated miRNAs (Fig. 9G). Then we predicted the binding lncRNAs of hsa-miR-7-5p and hsa-miR-877 through ENCORI database (Supplementary tables 4, 5), correlation analysis indicated hsa-miR-7-5p was the potential miRNA to have binding lncRNAs (Fig. 10A and Table 2), however has-miR-877 was not predicted to have target lncRNAs. MIR29B2CHG was prognosticated as an important regulator of hsa-miR-7-5p to UTRN. MIR29B2CHG was negatively correlated with hsa-miR-7-5p and positively correlated with UTRN (Fig. 10B,C). We next explored the mRNA expression of MIR29B2CHG in tumor and normal tissues from TCGA, the results showed that MIR29B2CHG was overexpressed in normal tissue (Fig. 10D). Survival analysis indicated a greater prognosis in individuals with a lower level of MIR29B2CHG expression (Fig. 10E). In above, MIR29B2CHG-hsa-miR-877-5p-UTRN was a potential regulatory upstream pathway of UTRN, which may contribute to endocrine therapy of breast cancer and immune checkpoint indication.

**Table 1:** miRNAs negatively related to UTRN.

Gene	miRNA	cor	*p* value	logFC	diff *P* val
UTRN	hsa-miR-93-5p	− 0.34291	0	1.041983	1.67E−28
UTRN	hsa-miR-106b-5p	− 0.34077	0	1.203527	1.51E−35
UTRN	hsa-miR-20a-5p	− 0.3109	0	0.466914	3.08E−05
UTRN	hsa-miR-17-5p	− 0.29358	4.16E−23	0.749094	3.91E−11
UTRN	hsa-miR-7-5p	− 0.28971	2.00E−22	1.09543	7.71E−28
UTRN	hsa-miR-107	− 0.28094	4.84E−21	0.660527	2.60E−21
UTRN	hsa-miR-15b-5p	− 0.26656	5.47E−19	0.87863	1.40E−16
UTRN	hsa-miR-877-5p	− 0.26377	9.99E−19	0.726616	1.85E−14
UTRN	hsa-miR-141-3p	− 0.26052	3.55E−18	3.044616	8.72E−50
UTRN	hsa-miR-19a-3p	− 0.25615	1.32E−17	0.871835	1.62E−12
UTRN	hsa-miR-103a-3p	− 0.25531	1.32E−17	0.621191	4.49E−18
UTRN	hsa-miR-200a-3p	− 0.25162	5.05E−17	2.526654	1.19E−43
UTRN	hsa-miR-15a-5p	− 0.24758	1.63E−16	0.886827	3.40E−27
UTRN	hsa-miR-98-5p	− 0.23382	7.40E−15	0.678773	3.36E−23
UTRN	hsa-miR-19b-3p	− 0.20584	8.51E−12	0.214352	0.055794

**Table 2:** lncRNAs negatively related to hsa-miR-7-5p and positively related to UTRN.

lncRNA	miRNA	cor	*p* value	logFC	diff *P* val
MIR497HG	hsa-miR-7-5p	− 0.2179	3.97E−13	− 0.75308	7.61E−58
MIR29B2CHG	hsa-miR-7-5p	− 0.24537	2.43E−16	− 0.17879	2.97E−08
LINC00663	hsa-miR-7-5p	− 0.2917	1.01E−22	− 0.31735	8.22E−23
MEG3	hsa-miR-7-5p	− 0.2794	6.59E−21	− 1.22696	3.32E−44
OIP5-AS1	hsa-miR-7-5p	− 0.25315	2.51E−17	− 0.37743	9.39E−16
MIR497HG	UTRN	0.069536	0.022004	− 0.75308	7.61E−58
MIR29B2CHG	UTRN	0.263964	1.23E−18	− 0.17879	2.97E−08
LINC00663	UTRN	0.135953	7.13E−06	− 0.31735	8.22E−23
MEG3	UTRN	0.357757	0	− 1.22696	3.32E−44
OIP5-AS1	UTRN	0.460638	0	− 0.37743	9.39E−16

## Discussion

The gene UTRN encodes the protein utrophin and is present in various tissues, with its highest abundance in skeletal muscle. It is an autosomal homologue of the dystrophin protein. Recent research has shown that high UTRN expression down-regulated the p38 and JNK/c-Jun molecular signaling pathways, thereby suppressing the proliferation of melanoma^14^. In our study, we also discovered that UTRN functions as a tumor suppressor, implying that reduced UTRN expression may contribute to tamoxifen resistance and alterations in the tumor immune microenvironment in breast cancer.

Tamoxifen is a representative selective estrogen receptor modulator (SERM), which hinders the progression of luminal subtype of breast cancer cells^16,17^. Tamoxifen serves as the most extensively used oral medicine in endocrine therapy, which is the initial line of treatment for hormone receptor-positive breast cancer^18^. However, a third of patients using tamoxifen end up developing drug resistance^19–21^. To identify novel treatment targets, it is crucial to investigate the mechanism of tamoxifen resistance. Ten genes changed between sensitive and resistant to tamoxifen treatment, according to our analysis of differentially expressed genes in two luminal subtypes of breast cancer patient samples treated with endocrine therapy and sensitive and drug-resistant cell lines (MCF7/MCF7R, T47D/T47DR) as well as clinical breast cancer sensitive and relapsed patient samples. Pan-carcinoma and prognostic analysis revealed low expression of UTRN contribute to breast cancer progression and endocrine therapy resistance. The results of our CCK-8 and RT-qPCR assays were consistent with this conclusion as well. Therefore, we speculate that UTRN might someday prove to be a promising therapy for the treatment of BRCA in light of these positive results.

The importance of UTRN in tumor development is due to the possibility that it contributes to immune regulation, particularly for the control of T cell activation and proliferation^22^. In GSEA analysis of UTRN, we found that the TGF-β signaling pathway, inflammatory response, and IL6-JAK-STAT3 signaling pathway were highly enriched in the group with high UTRN expression, which are all immunomodulatory related pathways. Tumor microenvironment checkpoint therapy is emerging as attractive targets for the developing of treatments. It has been reported that tumor microenvironment was involved in therapeutic resistance^23,24^. Previous study showed cancer associated fibroblasts (CAF) can induce tamoxifen resistance through MEK/ ERK signaling pathway^25^.

We extrapolated the singular effect of tamoxifen on cancer cells to the tumor microenvironment. Consequently, we analyzed the impact of UTRN on components within the tumor microenvironment and observed that UTRN has the potential to serve as an indicator for TME modulation. Considering the importance of immune cell infiltration in cancers^26–28^. For immune cell quantization in various UTRN expression patterns, we used the CIBERSORT algorithm. According to the analysis’s findings, the group with high UTRN expression had a smaller percentage of CD8 + T cells and a comparatively higher percentage of resting memory CD4 + T cells. Additionally, we discovered that UTRN expression was strongly inversely connected with CD8 + T cell counts and regulatory T cells, favorably correlated with resting CD4 + T cell counts. These findings imply that UTRN is crucial for controlling immunological invading cells in BRCA.

The ceRNA hypothesis states that in order for lncRNAs to operate as ceRNAs and control mRNA expression, they must compete with their corresponding miRNAs for the attention of mRNAs^29,30^. We identified UTRN’s target miRNAs. Hsa-miR-7-5p was discovered to have a predictive value for BRCA patients and to have a negative correlation with UTRN expression. We then used the same procedure to screen the upstream lncRNA of hsa-miR-7-5p. Previous studies revealed that the gene hsa-miR-7-5p could be useful for necroptosis and that it is expressed more frequently in neuroendocrine tumors^31,32^. MIR29B2CHG was shown to be downregulated in BRCA tissues, and knocking down its predicted promoter increased BRCA cell proliferation, migration, and invasion^33^. Earlier studies also have shown that MIR29B2CHG is one of the lncRNAs that can be used to predict CD8 T cell invasion and patient prognosis models in breast cancer^34^. These outcomes partially supported the viability of our analysis. We also admit that more experimental verification is required even if the ceRNA network of the UTRN was obtained by bioinformatics research.

In conclusion, we initially identified 10 genes associated with drug resistance by analyzing the tamoxifen treatment database. Subsequently, we conducted pan-cancer expression and prognosis analyses to investigate the potential involvement of the tumor suppressor gene UTRN in breast cancer management. Furthermore, we uncovered that the signaling pathways associated with UTRN may play a role in regulating the tumor immune microenvironment. Through CIBERSORT analysis, we established a link between high UTRN expression and immune microenvironment suppression. Additionally, we predicted the relevant ceRNA network upstream of UTRN and identified potential regulatory pathways. In summary, UTRN plays a role in breast cancer carcinogenesis, progression, and response to endocrine therapy. As a tumor suppressor gene, UTRN may serve as a valuable diagnostic tool for the detection and management of BRCA. However, our study has inherent limitations. We apologize for reaching the conclusion that UTRN plays a significant regulatory role in the immune microenvironment solely through database analysis and we lack experimental data to substantiate this conclusion. However, this limitation also guides the direction for our future research endeavors.

## Conclusion

This study is the first to show that BRCA, particularly tamoxifen-resistant BRCA, exhibits a decrease in UTRN expression. Our experiments have also confirmed that knocking down UTRN reduces tamoxifen sensitivity in HR + breast cancer cells. High UTRN expression correlates with a favorable prognosis, as indicated by various online databases. Additionally, multivariate Cox regression analysis highlights UTRN as a critical predictive biomarker for breast cancer patients. Furthermore, we observed a significant association between UTRN and immune infiltration. Moreover, we explored the probable upstream-regulated ceRNA network of UTRN. As a result, this study provides novel insights into understanding the crucial role of UTRN. Consequently, low UTRN expression may serve as a valuable biomarker in the diagnosis and treatment of breast cancer patients associated with immune infiltration.

### Supplementary Information

Supplementary Information 1.

Supplementary Figure 1.

Supplementary Figure 2.

Supplementary Legends.

Supplementary Table 1.

Supplementary Table 2.

Supplementary Table 3.

Supplementary Table 4.

Supplementary Table 5.

Supplementary Legends.

## Data Availability

The Gene Expression Omnibus database (GEO, https://cancergenome.nih.gov) and The Cancer Genome Atlas Program (TCGA) repository (TCGA, https://www.cancer.gov/ccg/research/genome-sequencing/tcga) contain the datasets analyzed in this investigation. The corresponding author can provide the datasets used and analyzed for this study upon reasonable request. This published article and its supplemental information files contain all of the data created or analyzed during this investigation.
