# Supplementary figures and images for: UTRN as a potential biomarker in breast cancer: a comprehensive bioinformatics and in vitro study
Source: Sci Rep. 2024 Apr 2;14:7702. doi: 10.1038/s41598-024-58124-5 (PMC10987506; doi:10.1038/s41598-024-58124-5)

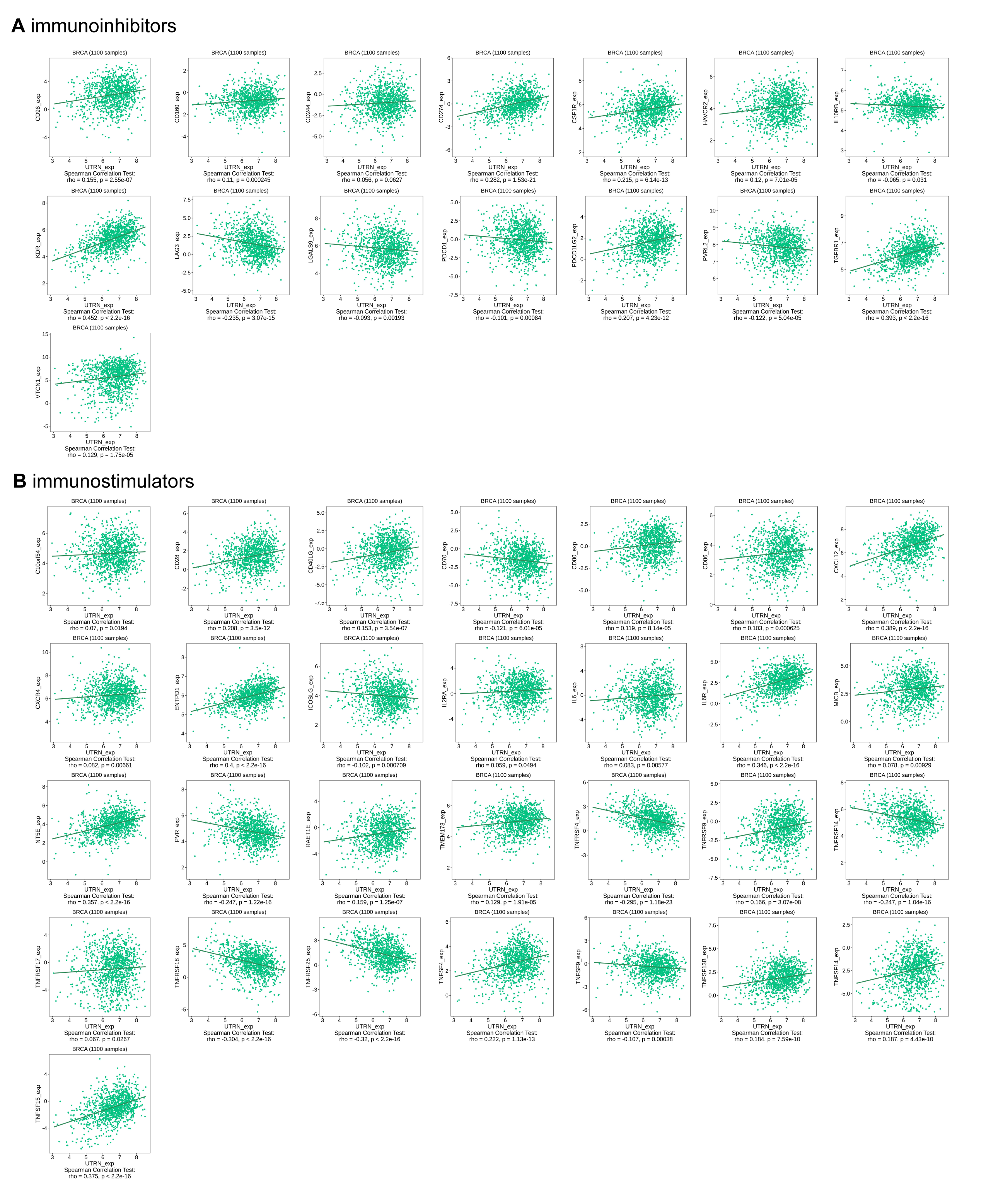

Supplement: Supplementary file 2 — Supplementary Figure 1. [file 41598_2024_58124_MOESM2_ESM.tif]

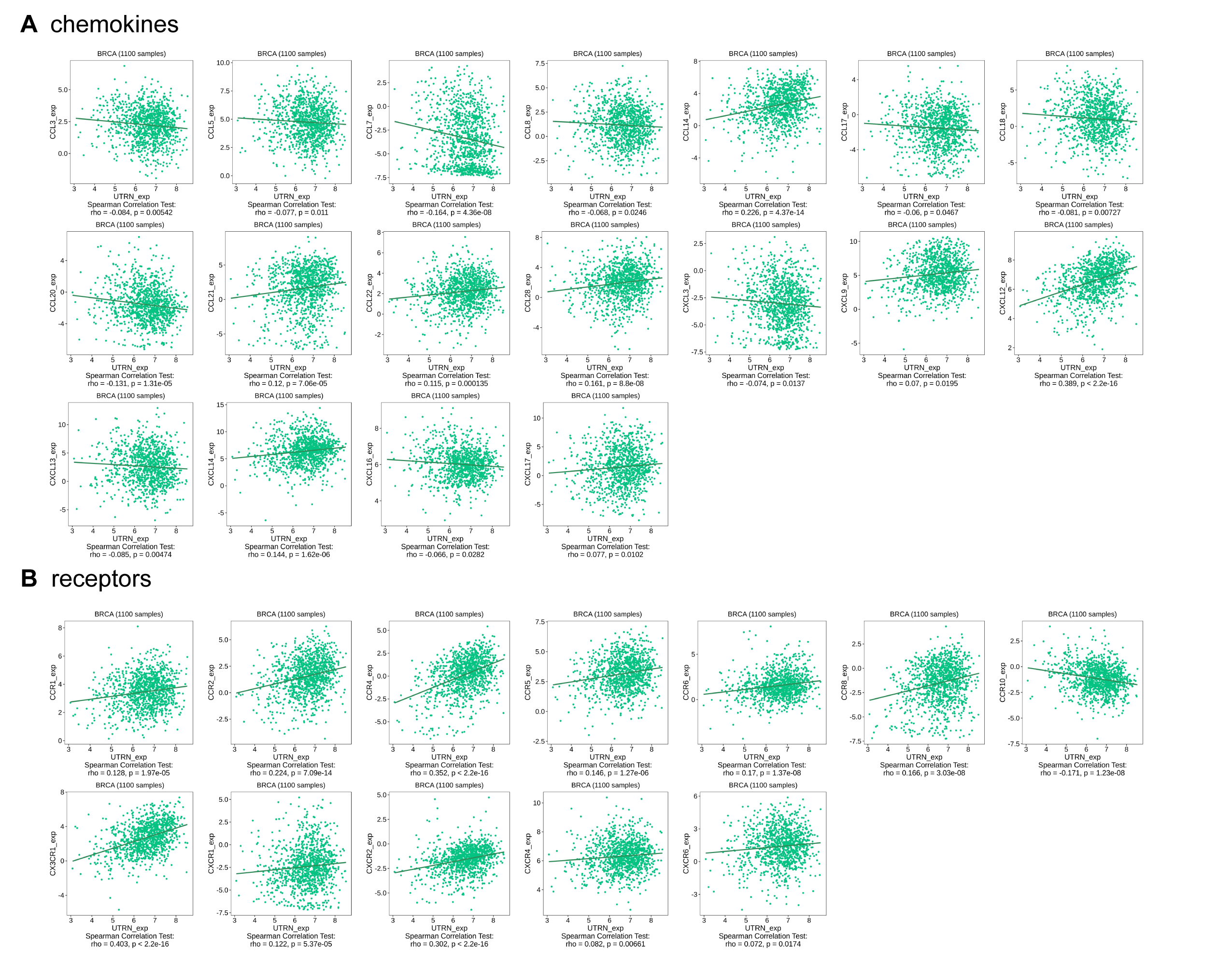

Supplement: Supplementary file 3 — Supplementary Figure 2. [file 41598_2024_58124_MOESM3_ESM.tif]
