# Supplementary material for: UTRN as a potential biomarker in breast cancer: a comprehensive bioinformatics and in vitro study
Source: Sci Rep. 2024 Apr 2;14:7702. doi: 10.1038/s41598-024-58124-5 (PMC10987506; doi:10.1038/s41598-024-58124-5)
Supplement: Supplementary file 4 — Supplementary Legends. [file 41598_2024_58124_MOESM4_ESM.docx]

Supplementary figure legends

Supplementary Figure 1. The expression of UTRN is associated with immunoinhibitors and immunostimulators in breast cancer. (A) Correlation between UTRN expression and immunoinhibitors in breast cancer available at TISIDB database. (B) Correlation between UTRN expression and immunostimulators in breast cancer available at TISIDB database. Color images are available online (Spearman, p<0.05 regarded statistically significant).

Supplementary Figure 2. The expression of UTRN is associated with chemokines and receptors in breast cancer. (A) Correlation between UTRN expression and chemokines in breast cancer available at TISIDB database. (B) Correlation between UTRN expression and receptors in breast cancer available at TISIDB database. Color images are available online (Spearman, p<0.05 regarded statistically significant).
