# Supplementary material for: UTRN as a potential biomarker in breast cancer: a comprehensive bioinformatics and in vitro study
Source: Sci Rep. 2024 Apr 2;14:7702. doi: 10.1038/s41598-024-58124-5 (PMC10987506; doi:10.1038/s41598-024-58124-5)
Supplement: Supplementary file 5 — Supplementary Table 1. [file 41598_2024_58124_MOESM5_ESM.docx]

Description about GSE datasets

|  | Sequencing technique | Sequencing platform | Samples number | Sample types |
| --- | --- | --- | --- | --- |
| GSE9893 | Expression profiling by array | GPL5049 | 155 | Primary tumors |
| GSE159968 | Expression profiling by array  Non-coding RNA profiling by array | GPL20115 | 9 | MCF-7, LCC2 and LCC9 cells |
| GSE125738 | Expression profiling by high throughput sequencing | GPL20795 | 6 | T47D and T47D-TR cells |
