# Supplementary material for: UTRN as a potential biomarker in breast cancer: a comprehensive bioinformatics and in vitro study
Source: Sci Rep. 2024 Apr 2;14:7702. doi: 10.1038/s41598-024-58124-5 (PMC10987506; doi:10.1038/s41598-024-58124-5)
Supplement: Supplementary file 10 — Supplementary Legends. [file 41598_2024_58124_MOESM10_ESM.docx]

Supplementary table legends

Supplementary table1. Description about GSE datasets.

Supplementary table2. Clinical information in TCGA-BRCA dataset.

Supplementary table3. Predicted target microRNA genes from ENCORI database by correlation analysis.

Supplementary table4. Predicted binding lncRNAs of hsa-miR-7-5p from ENCORI database by correlation analysis.

Supplementary table5. Predicted binding lncRNAs of hsa-miR-877 from ENCORI database by correlation analysis.
